# Political economy of health in fragile and conflict-affected regions in the Middle East and North Africa region

**DOI:** 10.7189/jogh.12.01003

**Published:** 2022-08-13

**Authors:** Shadi Saleh, Fouad M Fouad

**Affiliations:** 1 Global Health Institute, American University of Beirut, Beirut, Lebanon; 2 Department of Health Management and Policy, Faculty of Health Sciences, American University of Beirut, Beirut, Lebanon; 3 Faculty of Health Sciences, American University of Beirut, Beirut, Lebanon

The Middle East and North Africa region (MENA), is an epicentre of many different types of conflicts [1]. Twelve countries are facing humanitarian crises with unfortunate results ranging from fragility to massive refugees and displacement. These ongoing conflicts have immense health effects, including direct consequences (trauma-related deaths and injuries) and a disruption of the health system leading to increased morbidity and mortality [2]; resulting in a fragile and conflict-effected state. This special issue aims to provide grounded research in the political economy of health in fragile and conflict-affected settings in the MENA region by shedding light on the health status of refugees in the region, policies of the regions’ health systems, and the role of the government and the private sector in providing adequate health care services.

Papers in this issue reveal inequitable and inadequate access to health care services, including disease prevention, treatment, and care, as well as financial protection for vulnerable populations residing in fragile and conflict-affected countries in the MENA. Refugees also face significant public health challenges such as poor mental health status, poor reproductive and maternal health status, wasting among children, increasing noncommunicable diseases (NCDs), and communicable diseases, in addition to long-lasting causalities and injuries. Refugee women have unmet sexual and reproductive health (SRH) needs, barriers to access SRH services, inadequate provision of maternal health services (including family planning), and intimate partner violence (IPV). Singh et al. shows a 24% and 47% increase in IPV among refugees correlating with financial dependence on their husbands, respectively in this series [3]. Another study by Deuba et. al., reveals an increase in IPV associated with an increased financial dependence on spouse [4]. The current series further highlights that malnutrition, lack of quality education, poor physical and mental health, and extreme poverty have long- term consequences on children refugees, pushing them into labour market at an early age and young girls into early marriage. Moreover, Al-Haj et al., highlights that refugees are more susceptible to various types of injuries, including traumatic brain injury (TBI), due to the harsh living and working conditions that they endure [5]. Al Haj et al., found that the leading cause of these injuries among refugees in Lebanon were falls (44%) and violence (10%) [5]. The series also highlights the inequitable distribution of the COVID-19 vaccine in Lebanon. Kaloti et al., showed that 89% of vaccine doses have been administered to the Lebanese population, whereas only 5% have been administered to registered Syrian refugees [6].

The issue illustrates the interlink between refugee status and health care-seeking behaviour and access in host countries. This dynamic is amplified by the fact that many of the host countries have rather fragile health systems that were not able to accommodate significant surge demands on their infrastructure. According to Saleh et al., fragmented health care systems and high out-of-pocket (OOP) expenditure on health are two examples of the challenges that face many of the health systems in the region [7]. In Lebanon, there is an unregulated dominance of the private sectors in providing and financing health care services that are targeted, hence different services are provided to each social group. On the other hand, Jordan faces a dual challenge of extending the overburdened health services to meet the increasing demand whilst continuing to strengthen their quality of service. Additionally, Jordan struggles with limited financing in the health care system making it difficult to provide equitable services. Protracted episodes of conflict, political, and economic instability have further disrupted the health system in many states in the MENA [7]. International organizations, such as United Nations High Commissioner for Refugees (UNHCR), the World Health Organization (WHO), and the United Nations Relief and Works Agency (UNRWA), play an important role in attempting to provide adequate health care services in fragile and conflict-affected regions in the MENA where health care systems are ruled by political and economic instability, resulting in weakened systems.

The pre-existing fragile situation of refugees and health system in conflict settings was aggravated following the COVID-19 pandemic outbreak. Fouad et al., highlight that the main public health priorities in the MENA today is to properly detect and respond to the COVID-19 pandemic [8]. The socio-economic impacts of the pandemic prolonged the existing vulnerabilities of the fragile public sectors in the region. For example, when a weak health care system (such as in Afghanistan, Iraq, and Syria) is faced with a crisis like the pandemic; it fails to respond adequately and accordingly to the national guidelines [8]. Fouad et al show that most of the responses to the pandemic in the MENA are context-specific and are determined by the states’ vulnerability, fragility, and duration of long protracted crisis. Most importantly, the article highlights the pivotal role that UN agencies and international organizations are playing, together with ministries of health to a lesser extent, in ensuring adequate and proper response to the COVID-19 pandemic as the countries, alone, are not able to sustain proper response with poor infrastructures, segmented health system, and unclear policies.

The profound public health consequences related to migration in fragile settings in the MENA not only affect vulnerable populations, but also the communities and health security of the entire region. As Filippo Grandi, at the Commissioner of the UNHCR stated, “If we ever needed reminding that we live in an interconnected world, the novel coronavirus has brought that home” [9]. It is evident that the ability of the current health systems to provide equable health care is impacted by the loss of human and institutional infrastructure, instability of states, ongoing conflicts, and uncertainty in economic burden and political will. Witnessing the devastation of the already weak health systems in the MENA following a pandemic, highlights the important role of powerful actors, such as governments, UN agencies, and international organizations to shape national health agendas. This includes the formation of social protection systems across the MENA in adaptation to their social-cultural realities, hence alleviating the fragility of health care systems.

This series advocates for a change in political and health policies to ensure the provision of universal health coverage (UHC) and to meet the shared sustainable development goals (SGD)’s on health that entitle every person a right to a minimum standard of health care. To ensure the achievement of UHC and SGD, governments must reassess their economic and social policies, legal frameworks, and health systems to ensure the inclusion of all individuals. There is a limited understanding of the incentives and interests that are shaping policymaking and implementation within the political and economic paradigm. This is particularly true among fragile and conflict-affected regions which have a pre-existing weak health care system, deteriorating economy, and political instability. Powerful actors, such as donors, governments, and the private sectors, shape the national health agenda and the health system in each region. Those actors are greatly influenced by the political will and economic strains of the region. This series of articles aid in exploring the impact of prolonged crises and conflict on health status and understanding the political and economic determinants that are affecting people’s health. This knowledge helps shape health systems, policymakers, and implementation in conflict settings where the health care system is devastated following persistent political and economic unrest.
